# Saphenous vein graft and nitric oxide: strategies to prevent graft failure and enhance patency in coronary artery bypass grafting

**DOI:** 10.3389/fcvm.2025.1745260

**Published:** 2026-01-08

**Authors:** Michele Dell’Aquila, Sotirios Prapas, Giorgia Falco, Shadi Abdalla, Branden Tejada, Meher Challagalla, Ignazio Condello, Joshua Newman, Omar Jarral, Stevan Pupovac, Ameerah Ali, Konstantinos Katsavrias, Augusto D’Onofrio, Carlo Zebele, Antonio Totaro, Vincenzo Labriola, Tulio Caldonazo, Hristo Kirov, Antonino Di Franco, Jordan Leith, Lisa Rong, Mohammed Rahouma, Derek Brinster, Alexander Iribarne, Frank Manetta, Nirav Patel, Robert Kalimi, Mario Gaudino, Antonio Maria Calafiore

**Affiliations:** 1 Northwell Health, Cardiovascular Institute, New York, NY, United States; 2 1st Division of Cardiac Surgery, Henry Dunant Hospital, Athens, Greece; 3 Department of Cardiothoracic Surgery, Weill Cornell Medicine, New York, NY, United States; 4 School of Medicine and Surgery, University of Insubria Varese, Varese, Italy; 5 Division of Cardiac Surgery, University of Rome “Tor Vergata”, Rome, Italy; 6 Department of Cardiac Surgery, Clinica Montevergine, Mercogliano, Italy; 7 Department of Medicine and Health Sciences “V. Tiberio”, University of Molise, Campobasso, Italy; 8 Department of Cardiothoracic Surgery, Jena University Hospital, Jena, Germany

**Keywords:** composite graft, coronary artery bypass graft (CABG), endothelial dysfunction, graft failure, graft patency, nitric oxide (NO), perivascular adipose tissue (PVAT), saphenous vein graft (SVG)

## Abstract

Nitric oxide (NO) is a central regulator of vascular homeostasis and a key determinant of saphenous vein graft (SVG) outcomes in coronary artery bypass grafting (CABG). Endothelial dysfunction, driven by altered shear stress, oxidative stress, and cardiovascular risk factors, impairs NO production and release, contributing to SVG thrombosis, intimal hyperplasia, and atherosclerosis. SVG harvesting technique, storage, and intraoperative handling affects endothelial integrity, inflammatory response, and vascular remodeling, influencing arterialization, long-term patency, and clinical outcomes. Preservation of perivascular adipose tissue (PVAT) during vein harvesting enhances NO bioavailability, reduces inflammation and oxidative stress, and supports graft adaptation. Internal thoracic artery (ITA) grafts provide durable patency, survival benefit, and NO-mediated vasoprotection, improving SVG function and mitigating maladaptive remodeling. Graft configuration further determines SVG adaptation. ITA-composite SVGs confer continuous NO exposure, promote arterial-like remodeling, and attenuate low shear stress. Optimal secondary prevention, including antiplatelet therapy, statins and lifestyle modifications further preserves endothelial function and reduces SVG failure. Targeting NO through surgical technique, graft configuration, and pharmacologic intervention represents a unifying strategy to enhance SVG performance, arterialization, and long-term outcomes, addressing the current limitation of SVG in CABG.

## Historical perspective on nitric compounds in cardiovascular medicine

Nitroglycerin (NG) was first discovered in 1847 by Ascanio Sobrero, who firstly noted that it caused severe headaches. Alfred Nobel, who visited Sobrero's laboratory in Paris in 1850, recognized NG potential and began experimenting with it, manufacturing dynamite (1).

The first medical use occurred in 1858 when Alfred Field became the first physician to use NG clinically, treating a woman with severe angina (1). Thomas Lauder Brunton, in 1871, was among the first to understand the biological mechanisms of NG, particularly its vasodilatory effects on the vessel's walls and the nitrate tolerance concept. His work culminated in the 1,885 publication in the “*Textbook of Pharmacology and Therapeutics*” detailing NG's effects and mechanisms (2).

The early 20th century saw further discoveries, such as Francois-Franck's identification of amyl nitrite as a coronary vasodilator in 1903, and a decade later, Cow's observations on the vasodilatory potency of sodium nitrite and amyl nitrite in sheep coronary vessels (3, 4).

Interest in nitric compounds diminished during the rest of the century, until in the 1970s pharmacologist Ferid Murad and his colleagues showed that nitrite-containing compounds stimulated guanylate cyclase, increasing cellular cyclic guanosine monophosphate (cGMP) and causing vascular relaxation (2). During the same period and independently, Robert Furchgott and John Zawadzki found that endothelial cells (ECs) play a role in arterial smooth muscle relaxation, linking this effect to the activation of guanylate cyclase by nitric oxide (NO) and nitrite-containing vasodilators. They discovered a molecule responsible for this phenomenon, which they named endothelial-derived relaxing factor (EDRF) (5).

In the 1980s, Louis Ignarro's team identified EDRF as NO, demonstrating that NG and amyl nitrite formed unstable S-nitrosothiols, stimulating cGMP and causing vascular relaxation. Ignarro's experiments confirmed that EDRF and NO activate guanylate cyclase via the same mechanisms (6). Similar findings were made by Salvador Moncada, although his contributions were overlooked when the 1998 Nobel Prize in Physiology and Medicine was awarded to Furchgott, Ignarro, and Murad (7).

Since then, scientific interest around NO increased and it has been recognized as a crucial signaling molecule, involved in vascular homeostasis but also in neurotransmission, immune response, and cellular respiration. In the cardiovascular system, NO is responsible for vasodilation, inhibition of vascular cell growth, monocyte adhesion and release of endothelin-1, prevention of platelet adhesion and aggregation, stimulation of platelet cGMP production and suppression of adhesion molecules and chemokines (3).

In cardiac surgery, NO has been considered mostly as a cardiac and vascular protector. In fact, inhaled NO has been used in perioperative management during cardiac surgery under cardiopulmonary bypass (4, 8). Nevertheless, NO plays a crucial role in coronary artery bypass graft (CABG) surgery and specifically, in conduits atherosclerosis, long-term patency, and graft failure.

The greater saphenous vein (SV) is the most frequently used conduit in CABG (9). In this review, we outline the central role of NO in the pathophysiology of saphenous vein graft (SVG) failure and provide NO-based insights into the endothelial mechanisms, clinical outcomes, and preventive strategies to reduce SVG failure and to enhance long-term patency. Critically, we appraise surgical and pharmacologic approaches aimed at enhancing NO bioavailability and prevent SVG endothelial dysfunction. Eventually, we propose NO-centered strategies for the SVG, which may guide clinical practice and future research.

## Endothelial cells dysfunction and the role of nitric oxide

The vascular endothelium lines the interior walls of blood vessels. ECs and the basal lamina form a hemocompatible surface, namely, the vascular intima. Healthy vascular endothelium regulates vascular tone, cellular adhesion, thromboresistance, smooth muscle cell proliferation, and vessel wall inflammation by producing and releasing vasoactive molecules. These molecules, including bradykinin, thrombin, prostacyclin, and other prostanoids, modulate ECs function locally and drive arterial remodeling systemically. Among them, endothelium-derived NO is the most potent regulator of vascular tone and vascular homeostasis (10).

NO is one of the ten smallest molecules in nature and it is synthesized from L-arginine by endothelial nitric oxide synthase (eNOS) in the presence of cofactors (11). Once formed, NO is rapidly converted to nitrate and nitrite in the blood within 1.8 milliseconds (12). There are three isoforms of nitric oxide synthase (NOS) that produce NO: endothelial (eNOS), neuronal (nNOS), and inducible (iNOS). Each NOS isoform has a distinct role in regulating vascular tone. eNOS and nNOS are typically present in healthy endothelial cells, and are known as constitutive nitric oxide synthase (cNOS), while iNOS is primarily expressed during inflammation and/or infection (13, 14). cNOS isoforms require elevations in intracellular calcium for activation and therefore generate low amounts of NO for short periods (seconds to minutes), whereas iNOS functions independently of calcium and sustain NO production for longer periods (hours to days) (15, 16). Thus, iNOS can generate NO at concentrations up to 1000-fold higher than cNOS, with eNOS typically producing 0.1–130 nM and iNOS often exceeding 1,000 nM (17).

In order to regulate vascular function, ECs have developed a sophisticated mechanotransduction system that converts physical forces into biochemical signals. Under normal conditions, the endothelium maintains vascular homeostasis slightly favoring vasodilation. To achieve this, NO produced by ECs is released and diffuses into vascular smooth muscle cells, activating guanylate cyclase and leading to cGMP-mediated vasodilation (18). In this biochemical process, physical forces represent crucial signals for the ECs. In fact, ECs sense hemodynamic changes through a mechanosensory network composed by ion channels, glycocalyx, cell extracellular matrix (ECM) adhesions, intercellular junctions, and the cytoskeleton. The mechanosensors activate signaling pathways to release NO and other vasoactive molecules such as bradykinin, adenosine, vascular endothelial growth factor, and serotonin in order to adjust vascular resistance and normalize blood flow. ECs are exposed to several mechanical forces: radial forces from intravascular pressure, tangential forces from cell-cell interactions and vessel vasomotion, and axial shear forces from blood flow friction against the vessel wall. Axial shear stress is the most significant of these forces responsible for NO release. Higher shear stress leads to increasing NO release from ECs (19–21).

Mechanical forces with a defined direction, such as high shear stress, induce only transient vascular signaling of pro-inflammatory and proliferative pathways, which become downregulated as these forces are sustained. In contrast, mechanical forces with undefined direction, such as low flow or oscillatory flow, cause sustained signaling of pro-inflammatory, pro-thrombotic, and proliferative cellular pathways (22, 23). This leads to vascular structural remodeling to minimize intracellular stress/strain changes and adaptive signaling, forming a feedback mechanism to maintain vascular homeostasis and try to provide atheroprotection. Thus, flow patterns can either inhibit or promote endothelial dysfunction and consequent atherosclerosis (19, 20).

Endothelial dysfunction marks the initial stage of atherosclerosis, and it is associated with numerous cardiovascular conditions. Indeed, endothelial dysfunction is commonly observed in the presence of cardiovascular risk factors (13, 24). According to Raddino et al., the most influential cardiovascular risk factors for endothelial dysfunction are age, hypertension, dyslipidemia, diabetes, cigarette smoking, obesity, hyperhomocysteinemia, and asymmetric dimethylarginine (ADMA) (24). Aging impairs vasodilation, promotes vascular remodeling, and increases oxidative stress (24). Hypertension elevates vasoconstrictor mediators, angiotensin II, and endothelin, while reducing NO bioavailability (24). Dyslipidemia disrupts NO production through oxidative stress and peroxynitrite formation (24, 25). Diabetes impairs NO synthesis and heightens oxidative stress due to hyperglycemia and insulin resistance (24, 25). Smoking reduces NO production and endothelial function through oxidative stress (24). Obesity leads to endothelial dysfunction via insulin resistance and pro-inflammatory adipokines (24). Hyperhomocysteinemia increases oxidative stress and reduces NO bioavailability, promoting inflammation (24). ADMA inhibits NO synthesis and increases oxidative stress, contributing to cardiovascular risk and endothelial dysfunction (24).

In addition, Li et al. has shown that these risk factors are associated with an increased eNOS expression rather than a decrease (26). The electron flow within NOS is tightly controlled, and any disruption can lead to the generation of superoxide (O_2_-) instead of NO, a phenomenon known as NOS uncoupling. The produced NO reacting with dioxygen molecules O_2−_, and $O_{2-}^{2}$, forms cytotoxic compounds such as hydrogen peroxide (H_2_O_2_). The excess H_2_O_2_ production enhances eNOS expression, while accelerated NO degradation through its reaction with O_2−_ forms ONOO−, which perpetuates eNOS uncoupling and enzyme dysfunction (27). Consequently, these processes result in impaired endothelium-dependent vascular relaxation and, in the long-term, lead to endothelial dysfunction (24, 25, 28).

Thus, endothelial dysfunction arises from the interplay of hemodynamic forces, oxidative stress, cardiovascular risk factors, leading to impaired NO signaling and vascular remodeling. Understanding these mechanisms is essential to contextualize the role of NO on the SVG in CABG.

## Saphenous vein graft and acting forces

Coronary artery disease (CAD), also known as ischemic heart disease, is a leading cause of mortality worldwide and is associated with 17.8 million deaths annually (29). In the United States only, 610,000 deaths annually (29). The management of CAD encompasses preventive measures, including lifestyle modifications and pharmacological treatments, as well as revascularization procedures. Revascularization is performed through percutaneous coronary intervention (PCI) and/or CABG (30). Despite the less invasive nature of PCI, CABG remains the gold standard for patients with complex multivessel coronary artery disease, left main disease, diabetes, or reduced left ventricular function, in accordance with American and European guidelines (31, 32). The two most utilized conduits for CABG are the left internal thoracic artery (LITA) and the SV (33).

In 1946, Vineberg pioneered the implantation of the LITA through a tunnel within the anterior myocardial wall. Since then, significant advancements have established LITA as the preferred graft for bypassing the left anterior descending (LAD) artery, due to its superior 10-year patency rate (>90%), improved survival rates, and higher freedom from cardiac events compared to other conduits (34). In 1962, Sabiston was the first to use a SVG as a sutured aortocoronary anastomosis to the right coronary artery (RCA) (35). Subsequent years saw a marked increase in the use of SVG, which remains the most employed conduit globally in CABG for all non-LAD coronary territories, used in 80%–90% of patients (36).

SVGs are favored for their availability, ease of harvesting, and perceived resistance during manipulation and anastomosis, as well as their reduced susceptibility to vasospasm. However, up to 10%–25% of all the SVGs occlude within the first year post-CABG surgery (37). The rate of SVG failure, defined as the complete occlusion of the graft, can reach 40%–50% at 10 years post-surgery (38–40). This high failure rate adversely affects the long-term outcomes of surgical revascularization, leading to increased episodes of angina or myocardial infarction, and necessitating repeat revascularization. Consequently, SVGs are associated with poorer long-term patency compared to arterial grafts, primarily due to the development of intimal hyperplasia and accelerated atherosclerosis, collectively referred to as vein graft disease, which ultimately leads to SVG failure (41).

SVG failure pathophysiology encompasses three distinct phases. The first phase, which leads to primary graft failure, is characterized by thrombosis occurring within hours to 12 months post SV grafting. Both intrinsic and extrinsic technical factors influence early SVG thrombosis. The grafted vein undergoes early stresses, including ischemia-reperfusion injury during harvest and storage, disruption of the vasa vasorum and nerves within the adventitia and perivascular adipose tissue (PVAT), and stresses before anastomosis, such as uncontrolled mechanical over-distension to assess for potential leaks and extensive graft handling during implantation. These exacerbate endothelial damage, negatively impacting the entire SVG (9, 42, 43).

The combination of these primary stress factors induces inflammation and the formation of reactive oxygen species (ROS), reduce eNOS expression due to endothelial denudation, and damage ECs and smooth muscle cells (SMCs). In addition, the exposure of ECM proteins on the luminal surface leads to the accumulation of fibrin and platelets, while the release of prothrombotic and pro-inflammatory mediators trigger thrombin formation and promotes leukocyte adhesion and infiltration into the vessel wall. This disruption in local hemostatic balance and the resulting hypercoagulable state are sustained by the expression and release of prothrombotic mediators, such as platelet-derived growth factor, transforming growth factor-β, fibrinogen, fibronectin, and von Willebrand factor. Consequently, thrombus formation occurs, potentially resulting in early and acute thrombosis of the graft (44).

The second phase and significant stressor for the SVG is the formation of intimal hyperplasia, which is the primary cause of intermediate-phase SVG stenosis and occlusion. ECs damage triggers the expression of various growth factors and cytokines, promoting excessive ECM deposition in the neointimal compartment. SMCs from the media of the vein and the grafted artery, along with fibroblasts from the adventitia, begin to proliferate and migrate towards the intima of the vein graft. These processes initially occur predominantly at the anastomosis sites but eventually spread throughout the entire vein graft (9, 42, 43).

Accelerated atherosclerosis and subsequent plaque rupture are the third and main causes of late-phase SVG failure. The formation of atheromatous plaques is facilitated by predisposing factors for atherosclerosis, such as high blood pressure, diabetes, and obesity, along with damage and pathophysiological alterations to the vein wall induced by highly proliferative SMCs and the expression and secretion of pro-inflammatory cytokines. Local cytokines and inflammatory mediators stimulate monocytes to infiltrate the neointimal layer and differentiate into macrophages. The uptake of circulating low-density lipoprotein (LDL) particles by macrophages leads to the formation of foam cells. The progressive growth of these atherosclerotic plaques results in the development of necrotic cores, caused by dying foam cells and increased cholesterol deposition, as well as intraplaque hemorrhage from leaky angiogenic neovessels. These factors together lead to plaque rupture and subsequent SVG occlusion (9, 42, 43).

**Figure 1:**
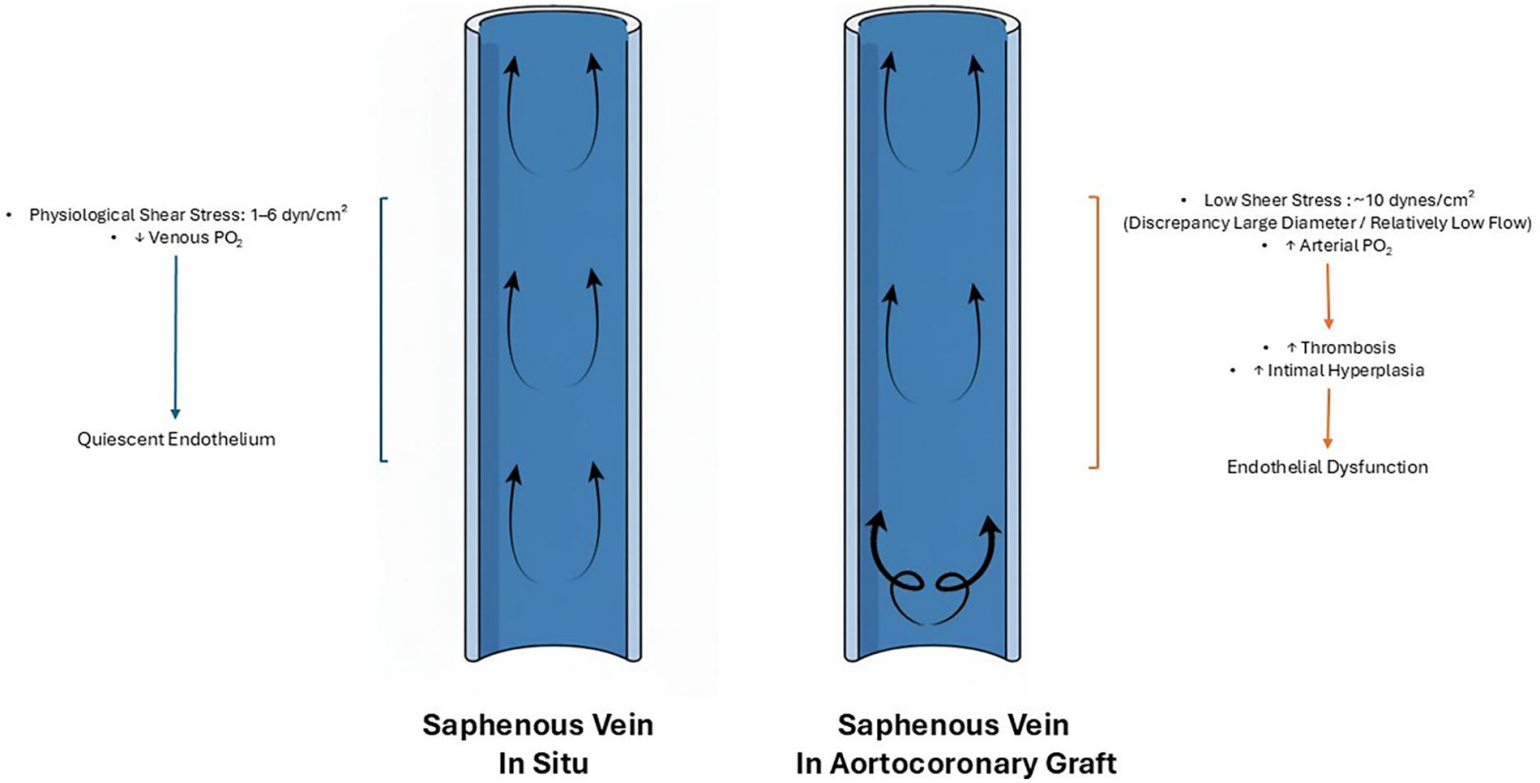
Saphenous vein *in situ* and aortocoronary graft. Hemodynamic changes in the saphenous vein *in situ* (left) versus the saphenous vein used as a aortocoronary graft (right). In situ, the saphenous vein experiences physiological shear stress (1–6 dyn/cm^2^), preserving endothelial integrity. In contrast, when used as aortocoronary grafts, the saphenous vein, due to the discrepancy between large diameter and relatively low flow, is exposed to pulsatile anterograde flow, proximal turbulent flow, and low shear stress (∼10 dynes/cm^2^) which lead to reduced nitric oxide production by the venous endothelial cells. This leads to increased risk of thrombosis and intimal hyperplasia, and eventually, to endothelial dysfunction of the saphenous vein.

Another critical factor for SVG failure is the exposure of the grafted vein to increased pressures within the arterial environment, known as arterialization. This process begins hours after implantation and exacerbates vein injury, promoting conduit remodeling (45, 46). Shear stress represents a fundamental variable in arterialization, influencing vein vascular morphology, remodeling, and endothelial function. In situ, venous shear stress typically ranges from 1 to 6 dyn/cm^2^, also previously reported as 0.2 dyn/cm^2^ by Lemson et al., and it is inversely proportional to vessel diameter (47, 48). SVG shows only a modest rise in wall shear stress when implanted into the arterial circulation. This is due to the SV larger diameter relative to the host artery, which impairs reaching shear stress levels observed in arterial conduits (49). Low shear stress under physiological conditions may be protective for venous ECs. Furthermore, *in situ*, venous ECs experience alterations in pressure (from ∼7 ± 1 mmHg supine to ∼76 ± 2 mmHg standing) and flow patterns due to orthostatic stress and temperature (50, 51). However, acute high shear stress in veins (12 dynes/cm^2^, consistent with arterial levels) can lead to formation of pro-inflammatory molecules by the ECs. Contrarily, prolonged high shear stress (10–15 dyn/cm^2^) on arterial ECs fosters an anti-inflammatory and anti-atherosclerotic environment, increasing the expression of eNOS and NO, facilitating vasorelaxation in response to elevated blood pressure and shear (Figure 1) (47, 52).

A proposed explanation for the differential shear stress response between arteries and veins is the selective activation of p38 in venous ECs. This occurs because shear stress triggers p38 signaling in veins but not in arteries, owing to absent mitogen-activated protein kinase phosphatase-1 (MKP-1) regulation (47). Upregulating MKP-1 in vein grafts could reduce pro-inflammatory signaling and facilitate the arterialization process under high shear stress (47).

A potential explanation for the differential response to shear stress by arterial and venous ECs may originate from embryonic vasculogenesis, resulting in two ECs types, with distinct morphologies, physiological responses, and phenotypes adapted to their specific vascular regions and functions (21, 47, 53).

Early research on ECs heterogeneity revealed that venous ECs, isolated from bovine tissue, were consistently larger, thinner, and more pleomorphic than arterial ECs, with notable differences in growth rate and protein biosynthesis. Protein markers specific to arterial and venous ECs are defined during vasculogenesis and remain as identifiers of vessel phenotype throughout life. Several studies have suggested that SV implantation into the arterial circulation results in a loss of venous ECs identity, but not a gain in arterial identity, suggesting an incomplete adaptation to the new hemodynamic environment (54–56). Furthermore, under arterial pO₂, SV showed a 2.8 fold higher intimal hyperplasia, 5.8 fold higher cell proliferation and 4 fold increase in ROS compared to fresh or venous pO_2_ (57). Veins perfused at pressures of 70 mmHg for 7 days showed significant neointima formation compared to those at 7 mm Hg (58). This endothelial change was linked to altered expression of molecular markers, such as matrix metalloproteinases (MMP-2 and MMP-9). This highlights the impact of hemodynamic forces on SVGs, demonstrating that arterial perfusion induces intimal hyperplasia without necessarily causing arterialization (58). However, further evidence is required to validate this assumption.

In summary, SVG failure requires three distinct phases: thrombosis, intimal hyperplasia, and accelerated atherosclerosis. All phases are amplified by hemodynamic stress of arterialization and the incomplete adaptation of venous ECs to the arterial environment. These concepts are fundamental to develop targeted strategies to enhance SVG patency and durability.

## Saphenous vein harvesting, endothelial damage and nitric oxide

SV harvesting is the first surgical step in preparing the vessel for use as a graft in CABG. The harvesting procedure inherently induces trauma, the extend of which varies according to the technique employed and may result in different degrees of vein dysfunction. Endothelial injury during harvesting initiates an inflammatory cascade, diminishes vasoactive capacity, and increases oxidative stress (59). Following graft implantation, inflammatory responses cause ECs apoptosis, which peaks approximately twenty-four hours postoperatively (60). This is followed by intimal hyperplasia and progressive wall thickening developing over weeks, with vascular remodeling persisting for up to six months. Therefore, preservation of endothelial integrity during harvesting is critical for the arterialization of SVGs, a process essential for venous adaptation to the coronary circulation and for achieving long-term graft patency.

Four techniques are employed for SVG harvesting: open conventional (OC), open no-touch (ONT), bridge, and endoscopic (61). The OC technique, widely used worldwide but less common in the United States, requires a longitudinal incision along the medial malleolus with complete dissection of the vein from surrounding tissues (62). In contrast, the ONT technique involves harvesting the vein as a pedicle through an open incision, preserving the vasa vasorum and perivascular nerves (63, 64). The bridge technique employs multiple small incisions along the vein course, minimizing wound size, bleeding, and infection risk (65). Finally, endoscopic vein harvesting is performed through a 2–3 cm incision, using systems such as Vasoview or VirtuoSaph and CO_2_ to create a dissection tunnel. The vein is then mobilized and harvested using electrocautery (66).

Several studies have investigated optimal strategies for SV harvesting in CABG, aiming at maximizing graft patency while reducing endothelial trauma thus, improving clinical outcomes.

In the PATENCY randomized controlled trial of 2,655 patients (mean age 61 ± 8 years; 22% women), Tian et al. demonstrated that the ONT technique significantly reduced graft occlusion compared with the OC method, both at 3 months [2.8% vs. 4.8%; odds ratio (OR) 0.57, 95% CI 0.41–0.80; *p* < 0.001] and 12 months (3.7% vs. 6.5%; OR 0.56, 95% CI 0.41–0.76; *p* < 0.001). Recurrence of angina was also lower in the ONT group at 12 months (2.3% vs. 4.1%; OR 0.55, 95% CI 0.35–0.85; *p* < 0.01), although rates of major adverse cardiac and cerebrovascular events (MACE) were similar. Notably, leg wound complications requiring surgical intervention were more frequent in the ONT group (10.3% vs. 4.3%; OR 2.55, 95% CI 1.85–3.52; *p* < 0.001) (67). The three-year follow-up confirmed the durability of these early benefits. Among the 2,655 randomized patients, 2,621 (99.4%) completed follow-up and 2,281 (86.5%) underwent CT angiography. The ONT technique remained superior, with significantly lower three-year SVG occlusion (5.7% vs. 9.0%; OR 0.62, 95% CI 0.48–0.80; absolute risk difference −3.2%) and consistent intention-to-treat results (6.1% vs. 9.3%; OR 0.63, 95% CI 0.51–0.81) (68). This trial showed that ONT technique reduced graft failure and related cardiac events by approximately one third to one half over three years, reinforcing its long-term advantage over the OC approach.

Zenati et al., in a randomized trial of 1,150 patients, part of the REGROUP trial, reported no significant differences in MACE between open and endoscopic vein harvesting at a median follow-up of 2.8 years [hazard ratio (HR) 1.12, 95% CI 0.83–1.51; *p* = 0.47] and at 4.7 years (23.5% vs. 21.9%; HR 0.92, 95% CI 0.72–1.18; *p* = 0.52). However, leg wound complications were less frequent in the endoscopic group [relative risk (RR) 2.26, 95% CI 0.99–5.15] (69, 70).

Operator experience has also been associated with SVG outcomes. Desai et al. reported that SV extracted by experienced harvesters (>30/month, >900 lifetime cases) had significantly less endothelial injury, particularly with open harvesting technique (71). Endoscopic techniques were associated with conduit injuries, including adventitial disruption, intimal tears, and intimal/medial dissections, although incidence was reduced with experienced operators. Patency resulted significantly lower in grafts with multiple dissections (67% vs. 96%, *p* = 0.05) (71). Similarly, Rousou et al. observed endothelial dysfunction in endoscopically harvested veins, attributed to caveolin displacement and reduced esterase activity, whereas Milutinović et al. reported better endothelial preservation with endoscopic harvesting at implantation, though no differences in clinical outcomes were observed at 1–2 years (72, 73).

Beyond harvesting technique, intraoperative graft handling is critical. The impact of storage solutions on SV's endothelium integrity is currently debated. Clinically, heparinized 0.9% saline or autologous whole blood (AWB) have been used (74). Both tend to damage the conduit's endothelium. Saline's pH is 5.5 while AWB becomes alkaline with a pH around 8.0, impairing endothelial and smooth muscle viability (75–77). Previous studies reported a less drastic injury with AWB than saline, though others found no difference between the two (76, 77). Functionally, AWB preserved conduits' contraction and relaxation better than saline (76, 78, 79). Buffered solutions provide physiologic pH and ionic balance, yielding superior endothelial preservation vs. AWB or saline (80). In PREVENT-IV, buffered solutions were associated with lower SVG failure and potentially improved outcomes compared to saline and AWB solutions (81). Endothelial damage inhibitor (EDI) is a buffered solution enriched with antioxidative, and eNOS-supporting components derived from the GALA formulation (glutathione, ascorbic acid, arginine) (82). In a small RCT to test EDI DuraGraft, SVGs stored in this solution showed significantly reduced wall thickness at 12 months compared with saline (83). *Ex vivo* studies on SVGs demonstrated that EDI decreases endothelial injury, lowers ROS and preserves eNOS and caveolin-1 expression, compared to saline, AWB, and standard buffered solutions (84–86). No comparative clinical studies have evaluated EDI vs. other buffer solutions for graft patency and clinical outcomes. Storage temperature may also influence SVG endothelial preservation, with limited evidence suggesting optimal protection at room temperature or 37 °C, while cooling to 4 °C may induce basal membrane separation and cellular deformation (87). Further studies on storage temperature are necessary.

Manual distension with a syringe to overcome spasm exposes the SV to pressures exceeding 300–400 mmHg. The usual exposure pressure for the SV is under 10 mmHg. Such supraphysiologic stress induces endothelial injury, medial apoptosis, and structural damage, with peak pressures up to 480 mmHg shown to disrupt all vessel wall layers (88–90). Intraluminal pressures exceeding 600 mmHg, causing endothelial denudation, smooth muscle apoptosis, and promoting intimal hyperplasia (91). Viaro et al. showed that pressures up to 200 mmHg preserved NOS activity, whereas fifteen seconds exposure to 300 mmHg led to endothelial loss and reduced eNOS expression (92). Pressure-controlled syringes may help in reducing this risk.

Currently, there is not enough evidence to demonstrate superiority in harvesting methods in terms of short and long-term cardiac outcomes. The priority remains ensuring conduit quality and long-term patency. As suggested by Sandner et al. (93), endoscopic harvesting is preferable in patients at high risk of leg wound complications, provided harvester experience is ensured. In contrast, open harvesting, ideally with the no-touch technique, remains optimal in patients at low risk of wound complications. SV should be stored in a buffer solution, high pressure distension should be avoided, and pressure controlled syringes should be used (93).

## Perivascular adipose tissue and nitric oxide-mediated endothelial protection

PVAT surrounds blood vessels and has emerged as an active vascular component, often referred to as a fourth vascular layer, the tunica adiposa (94). Previously regarded as a passive support and routinely removed to facilitate SV branches identification, PVAT has since been shown to exert important biological effects (94). SV graft patency is markedly improved when the vein is harvested atraumatically with PVAT preserved using the ONT technique. Long-term data confirm its superiority over conventional harvesting, with 16-year patency rates approaching those of the LITA (Patency ONT group 83% vs. Patency LITA group 88%) (95, 96). Randomized evidence by Dreifaldt et al. further supported this advantage, as ONT SV grafts show higher overall patency (91% vs. 81%, *p* = 0.046) and particularly favorable results in moderate stenoses (95% vs. 74%, *p* = 0.017) compared to RA grafts (97).

Mechanistically, the technique obviates the need for high pressure saline distension, prevents spasm by minimizing direct manipulation, and preserves a cushion of PVAT that provides both structural support and vasoprotective paracrine signaling (98, 99). Collectively, these findings indicate that PVAT preservation is a key determinant of long-term SV graft performance.

PVAT is phenotypically distinct from other adipose deposits. While adipocytes constitute most of its volume, they account for only one third of its cellular population. In fact, PVAT also contains nerve cells, endothelial precursor cells, fibroblasts, pericytes, macrophages, T and B lymphocytes and mesenchymal stem cells (94). Adipocytes within PVAT exhibit both white and brown adiposity features, highlighting its heterogeneity and adaptive potential. Importantly, PVAT varies by anatomical location, adiposity, and local pathophysiological environment. For instance, coronary PVAT promotes vascular injury through inflammation, oxidative stress, angiogenesis, and thrombosis, thereby accelerating atherosclerosis. Conversely, SV PVAT appears protective, especially when preserved with the ONT technique, and is associated with increased graft patency in patients undergoing CABG. Notably, the phenotype of SV PVAT resembles that of PVAT surrounding the ITA than that of coronary or aortic PVAT, a finding that may underlie the favorable outcomes of SV grafts harvested with intact PVAT (94, 100, 101).

**Figure 2:**
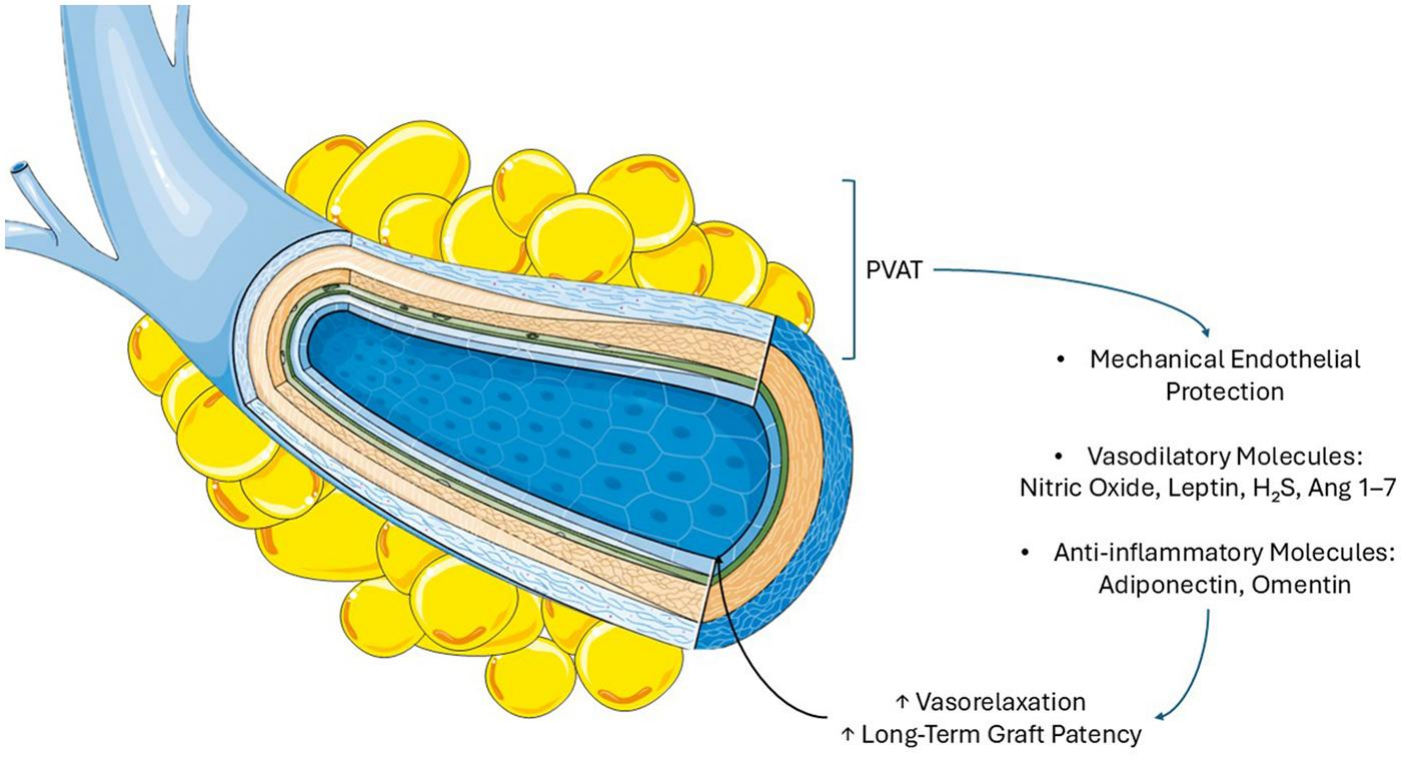
Saphenous vein graft and perivascular adipose tissue. Perivascular adipose tissue provides mechanical protection against pressure and distension injuries and secretes endothelial-protective molecules. Adiponectin reduces inflammation and enhances nitric oxide-mediated vasodilation, leptin promotes vasodilation and counteracts angiotensin II, omentin attenuates vascular inflammation, hydrogen sulfide relaxes smooth muscle by lowering intracellular calcium, and angiotensin 1–7 increases nitric oxide bioavailability and supports vasodilation. These molecules contribute to improve SVG long-term patency.

In his original article from 1968, one of the pioneer of the SVG, Favaloro advised dissecting the vein and removing the surrounding pedicle for an aortocoronary bypass (102, 103). However, through the years, PVAT demonstrated to function as a mechanical protector for the SV (Figure 2) (104). This role is linked to reduced metabolic inflammation and attenuated tissue remodeling (99). There is no mechanical barrier between the vessel wall and the PVAT, rather a fibrous layer separates adipocytes from adventitial cells. This arrangement facilitates direct communication among substances secreted by the PVAT and those released by the vessel wall (105). This leads to a complex, bidirectional communication between the vascular wall and its surrounding PVAT. Antonopoulos et al. demonstrated that vascular oxidative products such as 4-hydroxynonenal upregulate adiponectin expression in PVAT through peroxisome proliferator-activated receptor gamma (PPAR- *γ*) activation. In turn, PVAT-derived adiponectin enhances NO bioavailability by stimulating eNOS activation via PI3K/Akt phosphorylation and improving coupling through increased tetrahydrobiopterin (BH4) availability, while simultaneously reducing superoxide production (106–108). These findings suggest that oxidation products from the vascular wall may act as “rescue signals,” driving PVAT toward a protective phenotype as a local counter regulatory mechanism.

Recent evidence by Saito et al. indicates that PVAT expresses high levels of argininosuccinate synthase 1, sustaining continuous substrate availability for NO synthesis, together with expression of eNOS (109). ONT SVGs exhibited superior eNOS production compared with OC harvested grafts, largely attributable to PVAT (109). Compared with ONT harvesting, OC SVGs without PVAT, demonstrate greater vascular SMC injury and iNOS induction, although it is not clear if this rapid iNOS stimulation has harmful or protective effect on the graft (110). Segments with preserved PVAT contained higher eNOS protein and activity, were resistant to pressure induced injury, and maintained adventitial structures such as the vasa vasorum and perivascular nerves, key sources of NO (109). By contrast, OC harvesting disrupts the adventitia and vasa vasorum, promoting medial ischemia, vascular smooth muscle cell abnormalities, and neointimal hyperplasia (109, 110). Importantly, restoration of retrograde flow after anastomosis may reestablish vasa vasorum perfusion in ONT grafts. Preservation of PVAT and adventitial eNOS activity likely underlies the protective effect of the ONT technique, with downstream benefits including inhibition of platelet aggregation, reduction of early thrombosis, and facilitation of reendothelialization (109).

Besides NO, PVAT secretes other important adipokines and gaseous mediators with anti-contractile, vasodilatory, and anti-inflammatory properties. Adiponectin, abundantly secreted by PVAT, exerts vasoprotective effects by suppressing nuclear factor kappa-light-chain-enhancer of activated B cells (NF-κB)–mediated inflammation, reducing interleukin-6 (IL-6) and tumor necrosis factor-alpha (TNF-α), and promoting NO bioavailability via AMP-activated protein kinase (AMPK)—dependent eNOS activation and BH4 synthesis (108, 111–113). Leptin, localized in the PVAT of SV, similarly induces vasodilation through eNOS phosphorylation, AMPK activation, and angiotensin II antagonism, supporting the clinical superiority of ONT SVGs (98, 99, 114, 115). Omentin attenuates vascular inflammation by inhibiting thioredoxin-interacting protein/nucleotide-binding domain, leucine-rich–containing family, pyrin domain–containing-3 (TXNIP/NLRP3) signaling and oxidative stress, while circulating levels correlate with endothelial health (116–118). Hydrogen sulfide (H_2_S), another PVAT derived gasotransmitter, induces hyperpolarization of vascular SMCs by opening big-potassium (BK) channels and inhibiting L-type Calcium (Ca^2+^) entry, thereby lowering intracellular Ca^2+^ (94, 119). Finally, PVAT-derived angiotensin 1–7 enhances NO bioavailability via Mas receptor–dependent eNOS activation and BK channel stimulation (119, 120). Together, these mediators provide a biochemical and mechanistic rationale for the superior patency of ONT SVGs, where preserved PVAT actively contributes to long-term vascular homeostasis.

PVAT plays a fundamental role also in manual intraluminal distension. In the ONT technique introduced by Souza, PVAT, abolishes spasm without antispastic solutions, and avoids high pressure distension (121). PVAT provides mechanical protection against pressure injury and secretes paracrine anticontractile factors, thereby reducing vascular damage and improving long-term patency to levels comparable with the internal thoracic artery (99, 122–124). Removal of PVAT and reliance on high pressure distension, by contrast, result in immediate endothelial trauma and reduced long-term graft survival.

PVAT exhibits remarkable metabolic plasticity, rapidly adapting to systemic and local conditions. Nutritional status and adiposity strongly influence its phenotype (125). In both Asian and European cohorts, the protective effect of no-touch harvesting was lost in patients with elevated BMI (>27–30 kg/m^2^), suggesting an obesity driven phenotypic switch (67, 126). Mechanistically, excess PVAT may exert a restraint on vessel dilation and, through pro-inflammatory signaling, impair adaptive remodeling (127). Experimental studies further support this concept. In lean individuals, PVAT acquires a brown adipose tissue phenotype that mitigates post-surgical inflammation by releasing neuregulin-4 (NRG4), promoting macrophage polarization toward an anti-inflammatory phenotype, and increasing NO availability. By contrast, in obesity, PVAT shifts toward a pro-inflammatory state characterized by white adipocyte predominance, elevated IL-6, chemokine ligand 2 (CCL2), TNF-α, and leptin, and increased infiltration of pro-inflammatory macrophages (128–130). This environment suppresses PVAT browning, amplifies oxidative stress, and drives maladaptive vascular remodeling through enhanced vascular smooth muscle cell and mesenchymal stem cell proliferation and migration (125).

Despite these advances, the role of PVAT in postinterventional SV remodeling remains incompletely understood. Future work integrating single cell and spatial multiomics technologies is essential to dissect the heterogeneity of PVAT, delineate its molecular interactions with the vascular wall, and ultimately clarify its impact on long term SVGs outcomes.

## Nitric oxide from internal thoracic arteries benefits the saphenous vein

In CABG, the combination of an ITA and SVGs has represented the benchmark surgical strategy, although, after years, SVG failure remains a major limitation. Loop et al. in 1986 (131), demonstrated that patients receiving only SVGs had significantly worse long-term survival than those with an ITA graft, a finding subsequently confirmed in independent studies by Cameron et al. (132) and Boylan et al. (133). Long-term patency rates exceed 90% for both LITA and right internal thoracic artery (RITA) at 15 years (134), whereas SVGs exhibit failure rates of 15%–25% at 5 years and 40%–45% at 10 years (40, 135).

The ITAs can be harvested as a pedicled, including PVAT and endothoracic fascia, or skeletonized graft. Skeletonization provides greater conduit length, facilitates composite grafting, improves flow, and reduces sternal devascularization (136–138), though concerns about endothelial injury and long-term patency remain (137, 139, 140). A semi-skeletonized approach may offer a compromise, but data remain limited (141). Technical factors are also critical. Monopolar electrocautery may cause endothelial injury and vasospasm through heat transmission (>300 °C), whereas bipolar and harmonic devices minimize thermal damage (<80 °C), vasospasm and tissue charring, reducing need for clips (142, 143). Urso et al. in a randomized control trial and Keiser et al. in a large observational study suggest that harmonic skeletonization yields similar intraoperative flow and perioperative outcomes compared with electrocautery, without increasing risks of bleeding, conduit injury, sternal wound complications, or perioperative myocardial infarction (144, 145). Despite different techniques and instruments, ITAs' endothelium integrity is generally preserved during harvesting and manipulation.

The ITA exhibits unique endothelial properties that confer remarkable resistance to atherosclerosis and thrombosis. In patients with CAD, eNOS expression and NO release are markedly higher in the ITA endothelium than in carotid and radial arteries, impairing processes of atherogenesis such as lipoprotein oxidation, leukocyte trafficking, and vascular SMCs proliferation (14, 146–149). The antioxidant paraoxonase 2 (PON-2) is also abundantly expressed in the ITA, whereas its levels decline in carotids as atherosclerosis progresses (150). Platelet aggregates promote NO-mediated relaxation in the ITA, whereas they induce vasoconstriction in the SV. This underscores a protective role of platelet derived adenine nucleotides and purinergic signaling against thrombotic events after CABG in the ITA (151). Although NO bioavailability declines with aging due to impaired eNOS function, this can be partly restored by telomerase reverse transcriptase expression. Importantly, the ITA displays low telomere loss and absence of senescence associated *β*-galactosidase activity, suggesting resistance to age related endothelial changes that favor atherogenesis (152–157).

Shear stress profoundly influences ITA endothelium. Acute increases in flow enhance shear stress, inducing NO, prostacyclin, and endothelin-1 release to trigger vasodilation and augment blood supply (158). Sustained flow elevations remodel the artery, dilating it and increasing its diameter (159, 160). The native ITA environment is characterized by predominantly anterograde flow with minimal retrograde components, a pattern that activates cytoprotective and anti-inflammatory pathways (161). Consistently, endothelial- and flow- mediated responses are preserved in the ITA also in patients suffering from CAD and independent of age (162). By contrast, SVGs exposed to abrupt mechanical shear stress undergo inflammation and injury, whereas arterial endothelium resists this response, partly via shear induced MKP-1 expression that suppresses MAPK signaling (161, 163). ITA grafts also adapt to chronic flow changes: early post CABG they exhibit higher flow velocities than SVGs, which normalize at 1 year due to remodeling, while hyperemic flow reserve improves (164). Long-term imaging studies confirm that ITAs grafted for >10 years undergo structural adaptation, yet retain endothelial vasodilation (165). ITA's native and grafted hemodynamic environment elicits adaptive, anti-inflammatory, and cytoprotective responses that preserve vascular integrity and likely underlie its superior patency (166).

These ITAs responses may contribute to protect SVG. For instance, using the SV as a Y- or I- composite graft based on the *in situ* LITA offers several advantages over an aortocoronary SVG and provides outcomes comparable to the RITA composite graft.

The SV attached to the ITA is exposed to lower pulse pressure than when connected to the ascending aorta, as the ITA has lower diastolic and mean pressures (167). This reduces intimal hyperplasia, atherosclerosis, and endothelial injury. In addition, the SV conduit benefits from exposure to ITA derived vasoprotective mediators, such as NO (168, 169).

A composite SV graft also requires less conduit length than an aortocoronary graft, particularly when sequential anastomoses are used, enabling the use of SV segments with fewer valves. In fact, vein valves contribute to the development of stasis, flow disturbance, and pressure traps in the SV conduit segment and distal to the valve may further accelerate vein atherosclerosis (170). The SV has valves that are more frequently located right below the knee. Harvesting the SV from the upper or lower leg reduces this problem 171, 172).

Avoiding proximal anastomoses to the aorta also eliminates the need for aortic clamping, reducing the risk of embolic stroke and aortic dissection (171, 173).

In a randomized trial of CABG patients, SAVE RITA, Kim et al. showed that SV composite grafts were non-inferior to the right ITA in 1-year patency (97.1% vs. 97.1%, *p* = 0.958) and in MACE free survival at 1 and 4 years (97.3% and 94.4% vs. 98.2% and 91.1%, *p* = 0.597) when the SV was harvested using a minimal manipulation technique (174). A retrospective propensity matched CABG study by Hwang et al. further demonstrated comparable 5 year patency rates between SV and arterial composite grafts (93.9% vs. 89.1%, *p* = 0.246) (175). Compared to bilateral ITA grafting, composite SVGs preserve the RITA for future reinterventions and reduce the risk of perioperative morbidity, including deep sternal wound infection (174).

Nevertheless, studies evaluating SV Y-composite grafts based on the LITA have reported mixed results. Gaudino et al. analyzed 25 patients who received a composite SVG based on the LITA and recommended against the use of composite SVGs because they may steal flow from the LITA, leading to suboptimal LITA patency (72% at 2.5 years). Nevertheless, SVs of the composite grafts demonstrated a 96% patency. Issues were reported in the ITA used to revascularize a coronary artery with <70% stenosis (176).

Lobo Filho et al., using transit time flowmetry at baseline and during dobutamine stress, showed that both LITA and SV adapted appropriately to myocardial demand, and the presence of SV did not alter the physiological blood flow dynamics in the distal segment of the LITA (177).

Similarly, Glineur et al. demonstrated at 6 month follow-up that SV and RITA composite grafts had comparable hemodynamics in terms of pressure gradients at baseline, hyperemia and fractional flow reserve (178).

A propensity matched observational study by Hwang et al. validated the safety of composite SVGs. A total of 483 off-pump CABG patients were included, and 103 composite SVGs were matched with 103 RITA or right gastroepiploic composite grafts. No differences was found in survival, cardiac death, or graft patency up to 12 years, with SV harvested by the ONT technique (179).

The SAVE RITA trial corroborated these results. In 224 patients, SV and RITA Y-composites showed similar 10 year occlusion rates (6.9% vs. 3.4%, *p* = 0.21), equivalent survival (*p* = 0.75) and freedom from cardiac death (*p* = 0.16) (180).

Paterson et al. also reported favorable outcomes in 92 patients with ITA-based SV I-composites, with 89% 10 year freedom from SV occlusion and improved patency in grafts supplying multiple coronary targets (181).

Finally, Katsavrias et al., studied retrospectively 928 patients undergoing SVG to the RCA, inflow was provided either by the RITA (I-graft, *n* = 546) or the ascending aorta (Ao-graft, *n* = 382). After propensity matching of 306 patients per group and a median follow-up of 8 years, early outcomes were comparable, but the I-graft group showed superior 10-year survival (90.0% vs. 80.6%, *p* = 0.016) and freedom from MACE (81.3% vs. 64.7%, *p* = 0.021). Coronary CTA in a subset (*n* = 132) demonstrated higher 10-year patency with RITA inflow (82.8% vs. 58.8%, *p* = 0.003) and smaller SVG lumen diameter (2.7 vs. 3.4 mm, *p* < 0.0001). These findings suggest that continuous NO delivery from the RITA may enhance SVG performance, allowing it to function more like an arterial conduit.

These results were confirmed by Prapas et al. who studied 699 patients ≤75 years between 2000 and 2018 who underwent CABG with a single SVG directed to the RCA, using either the RITA stump as inflow (I-graft, *n* = 358, 51.2%) or the ascending aorta (Ao-graft, *n* = 341, 48.8%). After propensity matching, 272 patients were analyzed per group, with a median follow-up of 88 months (IQR 65–93). Early outcomes were comparable, but long-term survival favored the I-graft strategy (10-year survival: 90.6% vs. 78.2%, *p* = 0.0266), as did freedom from MACE (85.2% vs. 69.9%, *p* = 0.0179). At follow-up, SVG patency was significantly higher with I-graft inflow (81.6% vs. 50.7%, *p* < 0.0001), albeit with a smaller lumen diameter (2.7 ± 0.4 vs. 3.4 ± 0.6 mm, *p* < 0.0001). These findings suggest that SVG to the RCA shows superior patency and outcomes when supplied by the RITA rather than the ascending aorta (182). In summary, the ITAs demonstrates superior biological and hemodynamic properties in CABG, ensuring long-term patency and survival benefit, conferring NO-based vasoprotective effect when used as the inflow source for SVGs.

## Graft configuration using the saphenous vein graft

Graft configuration is a major determinant of patency and long-term outcomes. Traditionally, SVGs are anastomosed to the ascending aorta, where they are exposed to higher pressure, pulsatile flow, and circumferential strain of 10%–15%. In this setting, shear stress increases modestly (10–24 dynes/cm^2^) and long-term values typically remain <10 dynes/cm^2^ (183, 184). Isobe et al. reported shear stress values of 2.1 ± 0.3 dynes/cm^2^ in posterolateral branches and 3.6 ± 0.6 dynes/cm^2^ in the LAD for SVGs, compared with 13.8 ± 1.1 dynes/cm^2^ for LITA-LAD grafts (185). Similar findings were confirmed by Shimizu et al., who observed shear stress of 5 ± 2 dynes/cm^2^ at 1.5 years after surgery, irrespective of coronary stenosis severity (186). Most shear stress values registered remain below the 10 dynes/cm^2^ threshold (53, 187, 188). Thus, SVGs exposed to the aortic circulation experience similar shear stress to their native venous environment. These shear stress values remain in the atheroprone range.

Low shear stress downregulates eNOS expression, impairing endothelial-dependent vasodilation and reducing NO bioavailability (189–193). This deficiency promotes vasospasm and maladaptive remodeling in aortocoronary SVGs. By contrast, when the SVG is connected to an ITA, this adverse process is attenuated. Continuous NO release from the ITA may stabilize the SVG endothelium and protect the distal coronary territory from atherosclerosis (194). In this graft configuration SVG benefits from ITA derived NO, which stabilizes ECs and mitigates the adverse effects of the arterial environment. Furthermore, ITA would buffer the aortic pressure received directly by the SVG in an aortocoronary bypass. Collectively, these mechanisms support structural integrity and long-term adaptation of the graft.

Nevertheless, composite arterial-venous grafts are not without drawbacks. Mismatches in caliber and endothelial phenotype may predispose to flow competition and steal phenomena. Gaudino and colleagues reported reduced LITA patency distal to the LITA–SV junction, whereas Hwang et al. emphasized benefits of this strategy, including continuous exposure of the SVG to arterial NO, reduced conduit length, and avoidance of direct aortic anastomosis (176, 179). In the latter study, clinical outcomes were excellent, with 96.1% survival at a median follow-up of 128 months, although complete 10-year angiographic data were available for only 47.2% of patients, limiting the strength of conclusions (179). Furthermore, SVGs used as Y-grafts from the LITA have demonstrated comparable 10 year patency to RITA grafts (179). Angiographic studies confirmed that when connected to the LITA, SVGs undergo early lumen reduction and adaptive increases in shear stress within 1 year, without abnormal intima/media thickening. This remodeling shifts shear stress toward arterial values, promoting a more arterialized phenotype and long-term patency (179). Lobo Filho et al. similarly reported preserved patency at 94 ± 49 months with LITA–SVG Y-composites, compared with lower patency in aortocoronary SVGs used for RCA revascularization (195).

Alternative strategies include attaching the SVG to the RITA, particularly in cases of a calcified ascending aorta, where the SVG becomes indistinguishable from the RITA, adapting to its caliber (182, 196). These results support the concept that continuous NO release from arterial inflow enhances SVG adaptation, allowing it to function more like an arterial conduit and achieving outcomes comparable to complete arterial revascularization (197).

Taken together, these data suggest that NO supplementation, whether derived from the LITA, RITA and/or PVAT represents a common mechanistic pathway to improve SVG patency. This unifying hypothesis may explain why SVGs harvested with the ONT technique also demonstrate improved outcomes (197, 198).

**Figure 3:**
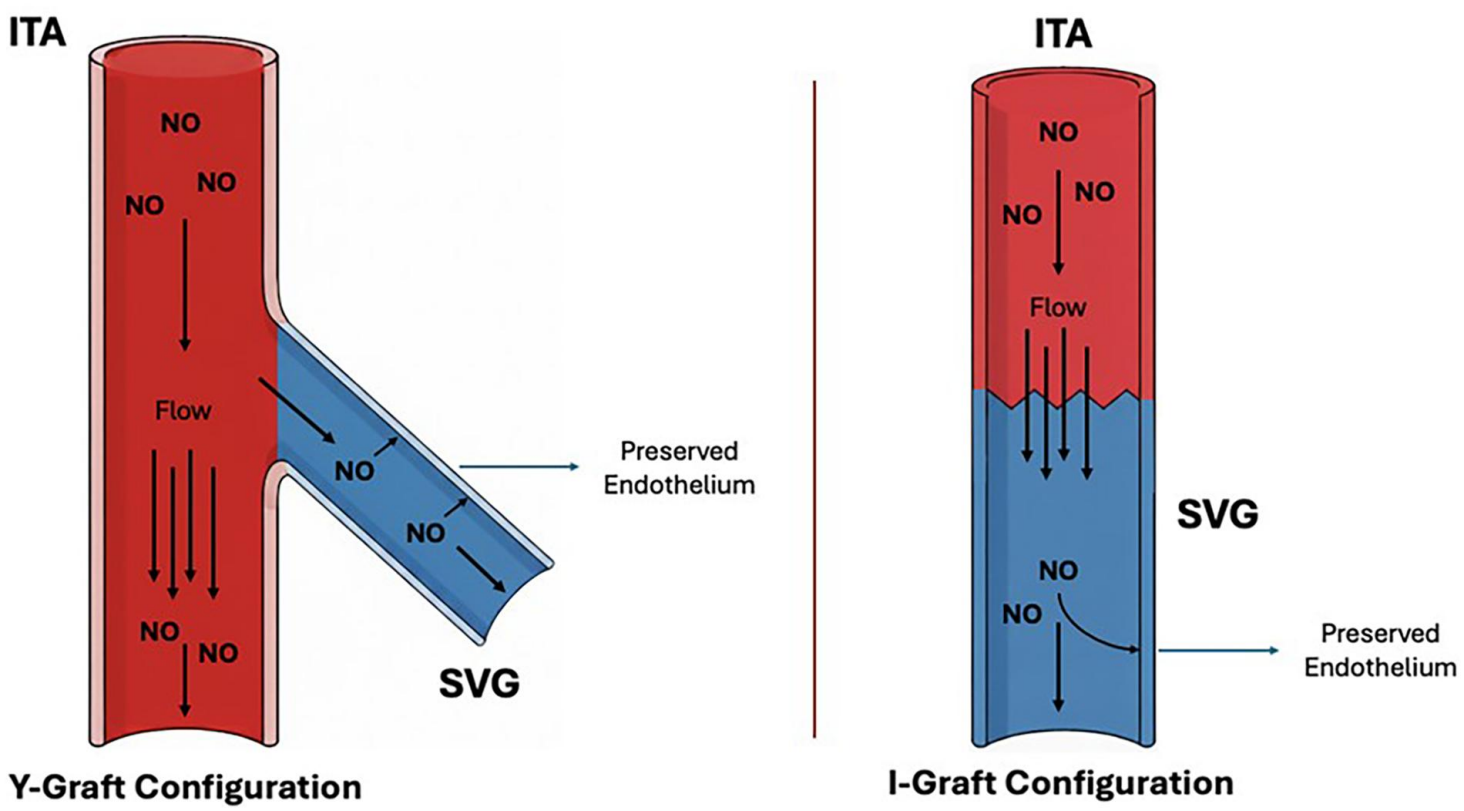
Saphenous vein graft and coronary bypass configurations. Y- and I-graft configurations using the internal thoracic artery and saphenous vein graft. In both configurations, inflow from the internal thoracic artery provides a continuous and superior source of nitric oxide and lower pulse pressure compared to an aortocoronary saphenous vein graft. These conditions preserve the endothelial integrity of saphenous vein graft, ensuring nitric oxide supply to the coronaries. Both configurations support vascular protection and long-term graft patency.

Current evidence indicates that maximizing NO bioavailability represents a unifying principle for improving SVG performance. Preservation of PVAT provides a local source of NO in aortocoronary grafts, while composite configurations with the ITA ensure continuous distal NO delivery (Figure 3). The combination of these two complementary procedures creates a synergistic vasoprotective environment that stabilizes the endothelium, attenuates maladaptive remodeling, and likely explains the superior patency and clinical outcomes observed with these strategies.

When PVAT is removed, as in OC or endoscopic SV harvesting, external support devices have been proposed to reduce SVG intimal hyperplasia. In their preclinical study, Jeremy et al. have shown that loose-fitting sheaths enhanced SVG remodeling and reduced neointimal hyperplasia in porcine models (199). In a phase I pilot study of 20 patients undergoing elective on-pump CABG, paired SVGs were tested with or without a Dacron stent (Extent). At six months, all 17 Extent-supported grafts were thrombosed, whereas all ITA-supported grafts and untreated SVGs stayed patent. Thrombosis was attributed to the rigidity, oversizing, or incomplete design of the Extent stent, which most likely caused postoperative kinking or distortion. These complications were not observed in previous porcine models. Despite the results, the authors sustained that flexible, nonrestrictive, biodegradable external support devices could have offered a safer option for future clinical use (200). This prompted the first clinical trial of the venous external support (VEST) device by Taggart et al., which showed that external stenting with a cobalt-chromium alloy modestly reduced diffuse intimal hyperplasia and improved lumen uniformity at 1 year, without altering SVG failure rates (201). Further studies found that the VEST does not lower rates of repeat revascularization or SVG occlusion, but it reliably reduces intimal hyperplasia and better preserves lumen geometry (202–204). The VEST trial follow-up is ongoing to elucidate the impact of external stenting on long-term SVG patency and clinical outcomes after CABG.

In summary, graft configuration strongly influences SVG adaptation and long-term patency. Aortocoronary SVGs are exposed to low, atheroprone shear stress and impaired NO bioavailability. Connecting SVGs to the ITA enhances NO-mediated endothelial stabilization, promotes arterial-like remodeling, and improves long-term outcomes. The VEST reduces SVG intimal hyperplasia and preserves lumen geometry, although follow-up data is necessary to evaluate long-term SVG patency and clinical outcomes.

## Secondary prevention of saphenous vein graft failure

Postoperatively, the prevention of secondary SVG failure is largely attributable to acute thrombosis and late atherosclerosis. The protective role of antiplatelet and lipid lowering agents in maintaining graft patency following CABG has been evaluated.

Early randomized clinical trials established aspirin as the cornerstone of therapy against SVG failure. In the Veterans Administration Cooperative Study, aspirin significantly reduced SVG occlusion compared with placebo at one year (15.8% vs. 22.6%, *p* = 0.029) (205). A subsequent meta-analysis of 17 randomized trials confirmed that low to medium daily doses of aspirin (100–325 mg), initiated within six hours after CABG, achieved maximal efficacy without increasing bleeding complications (206). By contrast, delayed aspirin initiation (≥24 h) conferred no benefit (207). For long-term maintenance, low dose aspirin (75–100 mg daily) is sufficient to suppress thromboxane A2 and address interindividual variability in response, though twice daily administration may be considered in the immediate postoperative setting due to increased platelet turnover following cardiopulmonary bypass (208). Although most individual trials were not powered for mortality outcomes, pooled data from 4413 patients and a total of 13,163 grafts, demonstrated that graft failure is a strong predictor of adverse cardiac events (OR 3.98, 95% CI 3.54–4.47) and mortality (OR 2.79, 95% CI 2.01–3.89), thereby indirectly underscoring aspirin's clinical benefit (209). Compared to aspirin, ticagrelor monotherapy did not improve SVG patency (210–212). In addition, factor Xa inhibitor rivaroxaban, either alone or in combination with aspirin, did not reduce graft failure as demonstrated in the COMPASS trial (213). By contrast, dual antiplatelet therapy (DAPT) has consistently shown superior efficacy over aspirin alone. A meta-analysis of 11 studies including 25,728 patients showed that aspirin combined with clopidogrel reduced SVG occlusion (RR 0.59, 95% CI 0.43–0.82), albeit at the cost of increased bleeding (RR 1.17, 95% CI 1.00–1.37) (214). A network meta-analysis of 20 randomized trials with a total of 4,803 patients confirmed improved graft patency with aspirin plus ticagrelor (OR 0.50, 95% CI 0.31–0.79) or aspirin plus clopidogrel (OR 0.60, 95% CI 0.42–0.86) compared to aspirin alone (215). Moreover, an individual patient level meta-analysis of four randomized trials (1,316 patients) demonstrated that aspirin plus ticagrelor reduced SVG failure rates (11.2% vs. 20%; OR 0.51, 95% CI 0.35–0.74), though at the expense of increased clinically relevant bleeding (22.1% vs. 8.7%) (216). To date, no head to head randomized comparisons between clopidogrel and ticagrelor exist, though ticagrelor is associated with more potent and consistent platelet inhibition (217, 218). Ongoing studies (ODIN, TOP-CABG) are expected to clarify whether short term or de-escalation DAPT strategies may optimize the balance between ischemic protection and bleeding risk (219, 220).

Lipid lowering therapy represents another cornerstone of secondary VGF prevention after CABG. The Post-CABG trial demonstrated that aggressive LDL-C reduction (<85 mg/dL) with lovastatin significantly decreased graft disease progression by 31% over a four year follow up period (221). Observational studies further suggest that achieving LDL-C < 100 mg/dL is associated with higher graft patency rates (222). The ACTIVE trial did not demonstrate superiority of high dose vs. moderate dose atorvastatin for SVG occlusion, although its interpretation was limited by small sample size (223). Beyond lipid lowering, statins exert pleiotropic effects including enhancement of endothelial function, increased NO bioavailability, and anti-inflammatory actions (224). More recently, proprotein convertase subtilisin/kexin type 9 (PCSK9) has emerged as a potential therapeutic target, with elevated circulating levels associated with SVG disease (225). The NEWTON-CABG CardioLink-5 trial randomized 782 post CABG patients with ≥2 SVGs to evolocumab or placebo on top of statin therapy. Despite achieving a 48.4% placebo-adjusted LDL-C reduction at 24 months, SVG disease rates were similar between groups (21.7% vs. 19.7%, *p* = 0.44). These results suggest that LDL-C lowering does not significantly impact early SVG failure mechanisms (226). Elevated HDL levels may help mitigate the development of SVG disease and lower the likelihood of adverse events after CABG. Jerzewski et al. showed that HDL <40 mg/dL was associated with higher SVG occlusion (6.8% vs. 4.0%, OR 3.2; *p* = 0.12) and more intimal hyperplasia (*p* = 0.10). HDL >60 mg/dL was associated with the least intimal hyperplasia (*p* = 0.01) (227).

Recent studies have explored strategies involving NO pharmacological strategy against SVG failure. In isolated SV segments from 30 patients, three NO donating aspirin adducts induced dose dependent relaxation, stimulated cGMP formation, and inhibited vascular SMCs proliferation, similar to sodium nitroprusside, whereas aspirin alone was inactive. This suggests that NO pharmacological therapy has potential to prevent thrombosis, vasospasm, and neointimal hyperplasia (228). In patients with type 2 diabetes mellitus, NO releasing aspirin restored impaired endothelium dependent vasodilation in SVGs, achieving maximal relaxation comparable to non-diabetic controls (56 ± 12% vs. 61 ± 11%), despite histological abnormalities such as intimal hyperplasia and endothelial degeneration (229). Complementing these findings, S-nitrosoglutathione induced vasorelaxation in SVs predominantly via cGMP dependent mechanisms, highlighting its potential to prevent graft spasm (230). Gene therapy also represents another innovative and viable approach. SVs transduced *ex vivo* with adenovirus vector encoding bovine endothelial eNOS showed endothelial and adventitial expression of recombinant eNOS, whereas control veins expressed only endogenous enzyme. Nitrite generation increased from 130.3 ± 19.9 to 1,420.0 ± 298.2 nM/mg (*n* = 3; *p* < 0.05), with maximal relaxation to calcium ionophore rising from 17.4 ± 7.4% to 32 ± 4.5% (*n* = 6; *p* < 0.05). Adenovirus-mediated eNOS transfer produced functional NO, suggesting potential to reduce early SVG thrombosis (231) Collectively, these studies support the concept that NO based therapies may enhance SVG function and reduce complications following CABG.

Lifestyle and behavioral factors are strongly associated with the risk of SVG failure. Active smoking nearly doubles the risk of graft occlusion at 1 year (HR 1.96, 95% CI 1.40–2.75) and accelerates progression of graft atherosclerosis (9, 232, 233). In 40 CABG patients (20 with type II diabetes, 20 without), diabetic SVG showed greater intimal fibrosis (1.95 ± 0.99 vs. 1.3 ± 0.8; *p* = 0.04) and ultrastructural changes including endothelial vacuolization and collagen accumulation. eNOS expression was significantly reduced in diabetics (endothelium: 2.10 ± 0.64 vs. 1.55 ± 0.68, *p* = 0.01; tunica media: 1.75 ± 0.63 vs. 1.2 ± 0.52, *p* = 0.007). These findings suggest diabetes promotes adverse structural and molecular alterations that may predispose to early graft failure (234).

In 276 hypertensive CABG patients followed for 3 years, higher postoperative BP (>130/80 mmHg) was strongly associated with SVG occlusion. Logistic regression confirmed BP as an independent risk factor (per patient OR 3.10, 95% CI 1.84–5.21; per graft OR 2.60, 95% CI 1.74–3.89; both *p* < 0.001). Use of ACEI/ARB or CCB was more common in the non-occlusion group, but no specific antihypertensive regimen correlated with graft patency (235).

Current guidelines recommend individualized, symptoms and risk-based surveillance post-CABG (236). Coronary computed tomographic angiography (CCTA) represents the primary modality for detecting graft failure, as duplex ultrasound has shown limited utility for coronary SVGs due to anatomic depth (236). CCTA offers high diagnostic accuracy, with reported sensitivity of ≈96% and specificity of ≈97% (237–239). Given the clinical importance of identifying early SVG failure, targeted follow-up should be prioritized in high-risk patients such as those with diabetes, multiple vein grafts, symptomatic ischemia, or suboptimal secondary prevention (9).

Thus, optimal secondary prevention and monitoring significantly improves graft patency and clinical outcomes, underscoring its central role in SVG management.

## Practical implications: nitric oxide-based recommedations for saphenous vein graft preservation

Endoscopic harvesting of the saphenous vein performed by an expert harvester is recommended in patients at high risk of leg wound complications. The open no-touch harvesting technique remains optimal in patients at low risk of wound complications.
Perivascular adipose tissue supports saphenous vein graft by mechanically protecting the vessel, preventing graft endothelial injuries, and secreting endothelial stabilizing molecules.
The saphenous vein should be stored in a buffered solution and high-pressure distension (>200 mmHg) should be avoided, using pressure syringes.
Connecting the saphenous vein in a Y- or I-graft to the internal thoracic artery shows improved long-term patency, while the saphenous vein aortocoronary graft shows reduced long-term patency.
Aspirin administration (100–325 mg) within six hours after coronary artery bypass grafting showed maximal efficacy in reducing saphenous vein graft occlusion, with reduced bleeding risk.
Dual antiplatelet therapy, despite increased bleeding risk, has shown superior efficacy over aspirin alone in reducing saphenous vein graft occlusion.
Secondary prevention strategies and clinical monitoring significantly improves saphenous graft patency and clinical outcomes.

## Conclusion

In surgical revascularization, the SV remains an indispensable conduit, yet SVG failure represents the Achilles' heel of CABG. Unlike arterial grafts, the SVG is a passive conduit, unable to accommodate hemodynamic stressors and prone to endothelial injury and maladaptive remodeling. NO has emerged as a unifying molecular target to prevent SVG failure, given its role in endothelial stability, vasomotor control, and long-term conduit adaptation.

Within this framework, NO-focused strategies could optimize SVG performance. Surgically, priority should be given to preserving the endothelial integrity and NO bioavailability through (1) ONT or expert-led endoscopic harvesting, (2) preservation of the PVAT derived from the native SV, (3) control of distension pressure (< 200 mmHg) through a pressure-controlled syringe (4) usage of a pH neutral (buffer) storage medium, and (5) ITA-based Y- or I-composite graft configurations. Pharmacological management should support the surgical efforts through (6) individualized antiplatelet therapy (favoring DAPT over aspirin, considering the bleeding risk) and (7) lipid-lowering therapy with statins. Secondary prevention should prioritize strict management of cardiovascular risk factors and close monitoring of individuals at increased risk for SVG failure.

Elucidating the impact of local NO bioavailability through randomized clinical trials focusing on SV harvesting, PVAT, conduit handling, grafting techniques, and pharmacologic interventions represent a crucial step in preventing SVG failure and optimizing graft patency. Future strategies to enhance NO, such as NO-donating medications or gene therapy, appear to be promising approaches to reducing SVG failure and improving long-term clinical outcomes in CABG patients.

## Glossary

ADMA: asymmetric dimethylarginine
AMPK: AMP-activated protein kinase
Ao: ascending aorta
AWB: autologous whole blood
BH4: tetrahydrobiopterin
BK: Big potassium (channel)
BMI: body mass index
CABG: coronary artery bypass graft
CAD: coronary artery disease
Ca^2+^: calcium ion
CCL2: chemokine ligand 2
cGMP: cyclic guanosine monophosphate
CI: confidence interval
cNOS: constitutive nitric oxide synthase
CO_2_: carbon dioxide
CCTA: coronary computed tomography angiography
EC: endothelial cell
ECM: extracellular matrix
EDI: endothelial damage inhibitor
EDRF: endothelial-derived relaxing factor
eNOS: endothelial nitric oxide synthase
HR: hazard ratio
H_2_O_2_: hydrogen peroxide
H_2_S: hydrogen sulfide
IL-6: interleukin-6
iNOS: inducible nitric oxide synthase
ITA: internal thoracic artery
L-arginine: levorotatory arginine
LAD: left anterior descending (artery)
LDL: low-density lipoprotein
LITA: left internal thoracic artery
MACE: major adverse cardiac and cerebrovascular events
MAPK: mitogen-activated protein kinase
MKP-1: mitogen-activated protein kinase phosphatase-1
MMP: matrix metalloproteinase
NF-*κ*B: nuclear factor kappa-light-chain-enhancer of activated B cells
NG: nitroglycerin
NLRP3: Nucleotide-binding domain, leucine-rich repeat-containing family, pyrin domain-containing 3
nM: Nanomolar
nNOS: neuronal nitric oxide synthase
NO: nitric oxide
NOS: nitric oxide synthase
NRG4: Neuregulin-4
OC: open conventional (vein harvesting technique)
ONOO^−^: peroxynitrite
ONT: open no-touch (vein harvesting technique)
OR: odds ratio
$O_{2}^{-}$: Superoxide
PCI: percutaneous coronary intervention
PI3K: phosphoinositide 3-kinase
PI3K/Akt: phosphatidylinositol 3-kinase/protein kinase B
PKB/Akt: protein kinase B
PON-2: paraoxonase 2
PP: pulse pressure
PPAR-*γ*: peroxisome proliferator-activated receptor gamma
PREVENT-IV: project of ex-vivo vein graft engineering via novel Technologies-IV trial
PVAT: perivascular adipose tissue
RA: radial artery
RCA: right coronary artery
RCT: randomized controlled trial
REGROUP: randomized endo-vein graft prospective trial
RITA: right internal thoracic artery
ROS: reactive oxygen species
RR: relative risk
SAVE RITA: saphenous vein vs. right internal thoracic artery
SMC: smooth muscle cell
SV: greater saphenous vein (native)
SVG: saphenous vein graft
TNF-α: tumor necrosis factor-alpha
TXNIP: thioredoxin-interacting protein
VEST: venous external support (device).
